# Development and Characterization of Synthetic Allotetraploids Between Diploid Species *Gossypium herbaceum* and *Gossypium nelsonii* for Cotton Genetic Improvement

**DOI:** 10.3390/plants14111620

**Published:** 2025-05-26

**Authors:** Sevara K. Arslanova, Ziraatkhan A. Ernazarova, Dilrabo K. Ernazarova, Ozod S. Turaev, Asiya K. Safiullina, Abdulqahhor Kh. Toshpulatov, Madina D. Kholova, Laylo A. Azimova, Feruza U. Rafiyeva, Bunyod M. Gapparov, Kuvandik K. Khalikov, Mukhammad T. Khidirov, Abdulloh A. Iskandarov, Davron M. Kodirov, Obidjon Y. Turaev, Salikhjan A. Maulyanov, Joshua A. Udall, John Z. Yu, Fakhriddin N. Kushanov

**Affiliations:** 1 Institute of Genetics and Plant Experimental Biology, Academy of Sciences of the Republic of Uzbekistan, Tashkent 111208, Uzbekistan; arslanovasevara87@gmail.com (S.K.A.); ziroat64@mail.ru (Z.A.E.); edilrabo64@gmail.com (D.K.E.); ozodturaev@gmail.com (O.S.T.); asiyasafiullina0996@gmail.com (A.K.S.); toshpolatovabduqahhor78@gmail.com (A.K.T.); mxolova107@gmail.com (M.D.K.); laylobio@gmail.com (L.A.A.); feruzarafiyeva25@gmail.com (F.U.R.); bunyodgapparov20@gmail.com (B.M.G.); quvondiqxaliqov87@gmail.com (K.K.K.); khidirov.tursunkilovich@gmail.com (M.T.K.); abdullohiskandarov4@gmail.com (A.A.I.); davronqodirov.083@gmail.com (D.M.K.); turaevobidjon@gmail.com (O.Y.T.); 2 Department of Genetics, National University of Uzbekistan, Tashkent 100174, Uzbekistan; 3 Research Institute of Plant Genetic Resources, National Center for Knowledge and Innovation in Agriculture, Tashkent 100180, Uzbekistan; 4 Department of Chemistry, National University of Uzbekistan, Tashkent 100174, Uzbekistan; smaulyanov@gmail.com; 5 United States Department of Agriculture (USDA)-Agricultural Research Service (ARS), Southern Plains Agricultural Research Center, College Station, TX 77845, USA; joshua.udall@usda.gov

**Keywords:** cotton, *Gossypium* L., wild species, *Gossypium herbaceum*, *Gossypium nelsonii*, interspecific hybridization, allotetraploids, SSR markers, genetic variation, fiber quality

## Abstract

Expanding genetic variability of cultivated cotton (*Gossypium hirsutum*) is essential for improving fiber quality and pest resistance. This study synthesized allotetraploids through interspecific hybridization between *G. herbaceum* (A_1_) and *G. nelsonii* (G_3_). Upon chromosome doubling using 0.2% colchicine, fertile F_1_C allotetraploids (A_1_A_1_G_3_G_3_) were developed. Cytogenetic analysis confirmed chromosome stability of synthetic allotetraploids, and 74 polymorphic SSR markers verified hybridity and parental contributions. The F_1_C hybrids exhibited enhanced resistance to cotton aphids (*Aphis gossypii*) and whiteflies (Aleyrodidae), with respective infestation rates of 5.2–5.6% and 5.4–5.8%, lower than those of *G. hirsutum* cv. Ravnak-1 (22.1% and 23.9%). Superior fiber length (25.0–26.0 mm) was observed in complex hybrids and backcross progeny, confirming the potential for trait introgression into elite cultivars. Phylogenetic analysis based on SSR data clearly differentiated *G. herbaceum* from Australian wild species, demonstrating successful bridging of divergent genomes. The F_1_C hybrids consistently expressed dominant *G. nelsonii*-derived traits regardless of the hybridization direction and clustered phylogenetically closer to the wild parent. These synthetic allotetraploids could broaden the genetic base of *G. hirsutum*, addressing cultivation constraints through improved biotic stress resilience and fiber quality traits. The study establishes a robust framework for utilizing wild *Gossypium* species to overcome genetic bottlenecks in conventional cotton breeding programs.

## 1. Introduction

Cotton is a globally significant cash crop, providing natural fiber for the textile industry. The genus *Gossypium* comprises four cultivated species and nearly 50 wild species [1,2,3]. Among the cultivated species, *G. arboreum* and *G. herbaceum* are diploids (2*n* = 2x = 26), while *G. hirsutum* (upland cotton) and *G. barbadense* (Pima cotton) are allotetraploids (2n = 4x = 52) [1,4,5]. Upland cotton accounts for approximately 95% of the global cotton production due to its high yield, early maturity, and ease of cultivation, whereas Pima cotton constitutes less than 2% [6,7,8]. Diploid cotton species are classified into eight genome groups (A, B, C, D, E, F, G, and K) [9], with tetraploid *G. hirsutum* and *G. barbadense* belonging to AD_1_ and AD_2_, respectively. These genome groups are geographically distributed, with D-genome types found in the Americas, A, B, E, and F genome types—in Asia and Africa, and C, G, and K genome types—in Australia [7,10,11].

Despite its global importance and widespread cultivation, upland cotton *G. hirsutum* (AD_1_ genome) continues to suffer from a relatively narrow genetic base, which limits its resistance to various biotic challenges, including insect pests and viral diseases. This limited variability underscores the need to explore novel genetic resources for enhancing resistance to economically damaging pests such as whiteflies (*Bemisia tabaci*) and aphids (*Aphis gossypii*), which are major concerns in many cotton-growing regions [1,5,12,13,14].

In addition to yield, key indicators of fiber quality in cotton include fiber length, strength, and fineness [2,6], as well as fiber color [15,16], all of which are crucial determinants of the economic value of cotton fiber. The fiber produced by most *G. hirsutum* varieties is typically white, with medium length and strength. Some wild species, however, exhibit non-traditional fiber pigmentation (such as tan, brown, or green), and in some cases, superior fineness and strength. These traits represent valuable genetic resources for diversifying fiber characteristics in *G. hirsutum* cultivars and enhancing efficiency in the textile industry.

Wild cotton species provide a rich reservoir of untapped genetic variation, particularly for traits related to pest and disease resistance. Among these, *G. herbaceum* (A_1_ genome) and *G. nelsonii* (G_3_ genome) have shown strong potential. *G. herbaceum* is known for its resistance to cotton leaf curl virus and multiple insect pests, including whiteflies, thrips, and aphids [17]. Likewise, *G. nelsonii* exhibits resistance to bacterial blight, *Verticillium dahliae*, aphids, and spider mites [18].

In addition to biotic stress tolerance, both species also carry valuable traits for abiotic stress adaptation, such as drought tolerance in *G. herbaceum* and salt or heat tolerance in *G. nelsonii*, which further demonstrates their value in improving stress resilience in cultivated cotton [12,13,14].

However, interspecific hybridization between cultivated and wild *Gossypium* species often encounters reproductive barriers, including reduced pollen viability, segregation distortion, and hybrid breakdown [19]. These challenges necessitate the implementation of cytogenetic analyses to understand meiotic irregularities, gamete viability, chromosome pairing, and genetic recombination [20]. Direct hybridization between wild species and *G. hirsutum* can be complex and time-consuming. To overcome these limitations, a novel approach involving the creation of synthetic allotetraploids by doubling the chromosome number of F_1_ interspecific hybrids was proposed [21]. This strategy facilitates gene transfer from wild diploid species to cultivated tetraploid cotton, effectively shortening the introgression process and simplifying the manipulation of wild cotton genomes.

Previous studies have demonstrated the successful generation of interspecific hybrids and allotetraploids in *Gossypium*, including combinations such as *G. arboreum* × *G. raimondii* [22], *G. hirsutum* × *G. sturtianum* [21], *G. arboreum* × *G. bickii* [23], *G. hirsutum* × *G. klotzschianum* [24], *G. herbaceum* × *G. mustelinum* [17], etc. Building on this foundation, the present study focuses on the development of synthetic allotetraploids derived from *G. herbaceum* and *G. nelsonii* to facilitate the transfer of valuable traits from these wild species into *G. hirsutum*.

This study aimed to enrich the genetic base of *G. hirsutum* by developing synthetic allotetraploids from *G. herbaceum* and *G. nelsonii*. This process involved generating interspecific hybrids between various *G. herbaceum* subspecies and *G. nelsonii*, followed by colchicine treatment of the F_1_ hybrids to induce chromosome doubling and create fertile allotetraploids (A_1_A_1_G_3_G_3_). The resulting allotetraploids were characterized morphologically, cytogenetically (assessing chromosome number and pollen viability), and molecularly (using SSR markers). Finally, these allotetraploids were hybridized with *G. hirsutum* cultivars to initiate the introgression of valuable traits from the wild species into cultivated cotton, ultimately contributing to the development of improved varieties with enhanced resistance to biotic and abiotic stresses.

## 2. Results

### 2.1. Fertility of Parental Lines and Interspecific Hybrids: Boll- and Seed-Setting

Obtaining valuable and unique breeding materials with economically important traits through interspecific hybridization and utilizing their genetic potential in breeding programs can enable the development of new, superior cotton varieties.

Productivity, determined by the number of bolls and seeds per plant, in interspecific hybrids of diploid cotton species with different genetic and phylogenetic backgrounds depends on the compatibility of their chromosomes and genes.

Observations revealed variation in productivity-related traits among *G. herbaceum* subspecies and Australian wild species. In our study, the number of bolls per plant in *G. herbaceum* subspecies ranged from 22 to 49, while complete seed-setting ranged from 13 to 18. In Australian wild species, the number of bolls per plant ranged from 41 to 45, and complete seed-setting ranged from 8 to 12 (Table 1).

The highest number of bolls per plant was observed in *G. herbaceum* subsp. *euherbaceum* A256, with an average of 49 bolls per plant. The complete seed-setting per boll was 91.0% (ranging from 75.0–100.0%), and the coefficient of variation was 8.9%.

Similar results were observed in *G. herbaceum* subsp*. pseudoarboreum* f. *harga*, with an average of 42 bolls per plant. The complete seed-setting per boll was 90.0% (ranging from 80.0–100.0%), and the coefficient of variation was 8.4%.

The wild *G. herbaceum* subsp*. africanum* and *G. herbaceum* subsp*. pseudoarboreum* showed low productivity, with an average of 28 and 22 bolls per plant, respectively. The average complete seed-setting per boll was 88.0% and 90.0%, respectively, with ranges of 86.0–100.0% and 85.0–100.0%, and coefficients of variation of 4.9% and 4.3%, respectively.

In Australian wild species, such as *G. australe*, *G. nelsonii*, and *G. bickii*, the average number of bolls per plant ranged from 41 to 45. The complete seed-setting per boll ranged from 93.0% to 95.0% (with a range of 86.0–100.0%), and the coefficient of variation was 4.4–6.6%.

To explore the potential of interspecific hybridization, 14 different cross-combinations were made between *G. herbaceum* subspecies (A_1_ genome) and Australian wild species (G genome), aiming to develop hybrids with valuable traits for use in genetics and breeding studies (Table 2).

Interspecific hybridization success varied considerably. Boll-setting rates of the hybrids in the recipient buds varied from 1.8% to 66.7%. The highest compatibility was noted between *G. herbaceum* subsp. *euherbaceum* (cv. A-256) and *G. nelsonii* (66.7%), while the lowest was observed between *G. herbaceum* subsp. *frutescens* and *G. nelsonii* (1.8%). The average seed-setting within the hybrid bolls also showed variation, ranging from 41.0% to 66.7%. Notably, crosses with *G. herbaceum* subsp. *pseudoarboreum* exhibited the highest pollen viability rates, reaching 86.4% with *G. nelsonii* and 85.0% with *G. bickii* (Table 2).

The initial hybridization trials demonstrated the potential for producing fertile offspring between distantly related *Gossypium* species with differing genomes (G and A_1_). This suggests that even phylogenetically distant species within the genus can be successfully interbred.

Typically, hybrids obtained from crosses between distantly related species are sterile. To obtain fertile hybrids, colchicine was applied before sowing the seeds in pots. The polyploid hybrids were obtained according to the scheme in Figure S1.

**Figure 1:**
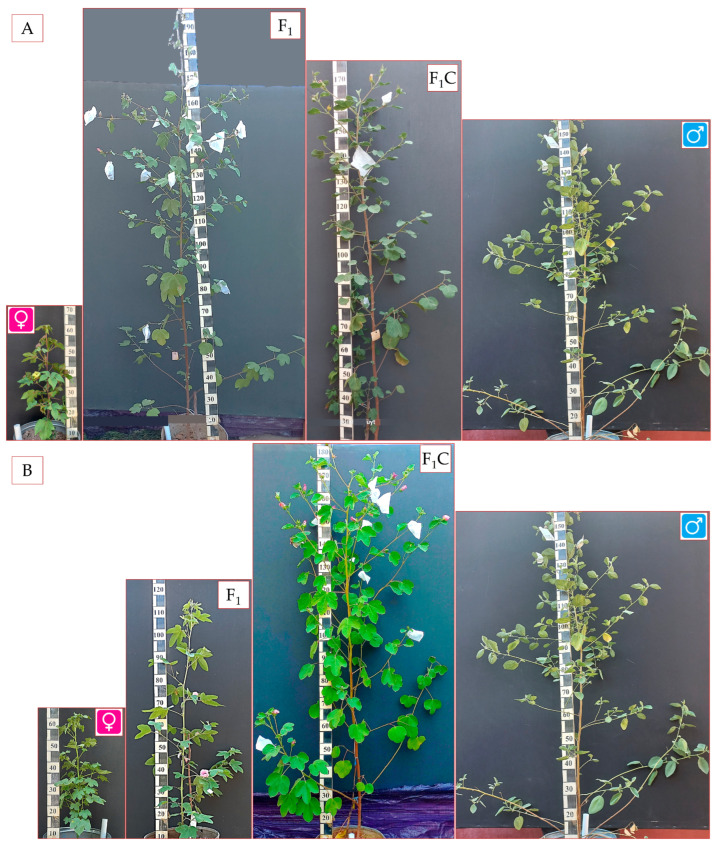
Diploid and allotetraploid hybrids with parental genotypes. (**A**) 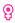
—diploid *G. herbaceum* subsp. *frutescens*; 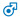
—diploid *G. nelsonii*; F_1_—diploid first generation; F_1_C—allotetraploid hybrid progeny of the first generation. (**B**) 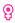
—diploid *G. herbaceum* subsp. *euherbaceum* (cv. А-256); 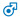
—diploid *G. nelsonii*; F_1_—diploid first generation; F_1_C—allotetraploid hybrid progeny of the first generation.

As shown in Table 2, four cross-combinations were unsuccessful, yielding no hybrids. In one combination, the seeds were not viable. Following colchicine treatment of the remaining nine combinations, only two—*G. herbaceum* subsp. *euherbacium* (cv. А-256) × *G. nelsonii* and *G. herbaceum* subsp. *frutescens* × *G. nelsonii*—successfully generated F_1_C hybrids (Figure 1). The other seven combinations failed to germinate, possibly due to seed coat thickness or other factors.

### 2.2. Inheritance of Morpho-Biological Traits in Interspecific Polyploid Hybrids

#### 2.2.1. Vegetative Growth Duration of Parental Lines and Polyploid Hybrids

To assess the inheritance of key traits in the interspecific polyploid hybrids, we analyzed the vegetative growth period, a crucial determinant of cotton maturity and adaptation to different growing seasons. Table 3 presents the vegetative growth duration (in days) for the parental lines (*G. herbaceum* subspecies and Australian wild species), as well as for the F_1_C and F_2_C polyploid hybrids. Among the *G. herbaceum* subspecies, subsp. *africanum* exhibited the longest vegetative period (142.9 ± 0.4 days), while subsp. *frutescens* had the shortest (101.2 ± 0.5 days). The Australian wild species, *G. australe*, *G. nelsonii*, and *G. bickii*, displayed relatively similar vegetative periods, ranging from 133.6 to 135.5 days.

The F_1_C allotetraploid hybrids derived from subsp. *frutescens* × *G. nelsonii* and subsp. *euherbaceum* (cv. A-256) × *G. nelsonii* exhibited vegetative growth durations of 127.5 ± 0.4 and 128.1 ± 0.5 days, respectively. This suggests a relatively uniform expression of early maturity traits in the F_1_C generation, likely attributable to partial dominance or complementary gene interactions. In the F_2_C generation, the subsp. *frutescens* × *G. nelsonii* hybrid showed a further reduction in vegetative period to 116 ± 1.05 days, while the subsp. *euherbaceum* (cv. A-256) × *G. nelsonii* hybrid maintained a similar duration to that of the F_1_C generation (127.9 ± 1.7 days). These results indicate possible segregation of early maturity-related traits in subsequent generations. Such findings are consistent with previous studies in *G. hirsutum* that have identified major QTLs and regulatory genes controlling flowering time and growth duration [5]. These traits may segregate in later generations and can potentially be selected through marker-assisted selection approaches for the development of early-maturing cultivars.

The coefficient of variation (V%) was generally low across all lines, indicating a relatively uniform vegetative period within each group. These findings demonstrate the heritability of vegetative growth duration and its potential for modification through interspecific hybridization and polyploidization. The observed differences in the vegetative period between the parental lines and the hybrids highlight the potential for selecting and developing cotton varieties with tailored maturity traits.

#### 2.2.2. Morphological Description of the F_1_C Allotetraploid Hybrids

**Figure 2:**
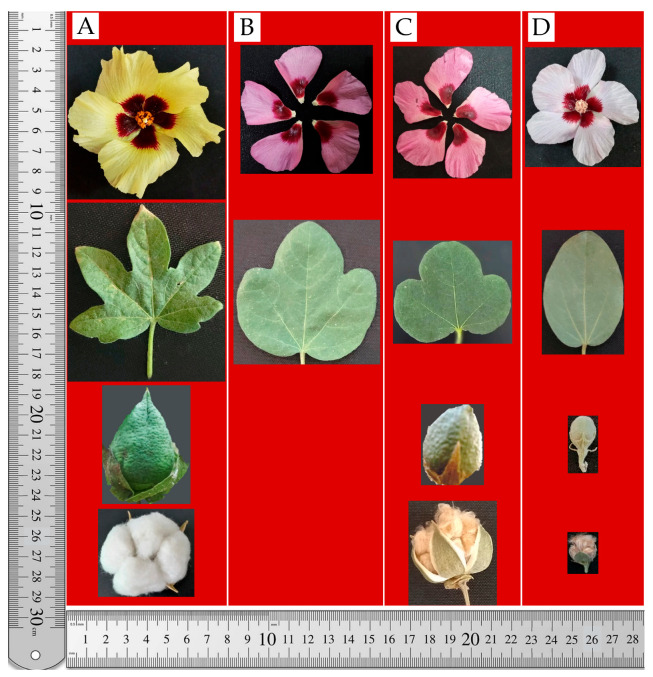
Morphological characteristics of cotton genotypes. (**A**) *G. herbaceum* subsp. *frutescens*—(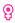) diploid maternal genotype; (**B**) diploid first generation—(F_1_) *G. herbaceum* subsp. *frutescens × G. nelsonii*; (**C**) allotetraploid hybrid progeny of the first generation—(F_1_C) *G. herbaceum* subsp. *frutescens × G. nelsonii*; (**D**) *G. nelsonii*—(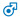) diploid paternal genotype.

The main stem of the *G. herbaceum* subsp. *frutescens* × *G. nelsonii* hybrids is erect and branched, with leaves arranged at medium density. The main stem height ranges from 180.0 to 190.0 cm, and it is green in color, strongly pubescent, with a weak anthocyanin blush. There are 28–30 nodes in total. Branching is sympodial, with the first sympodial fruiting branch forming at nodes 7–10. There are 2–3 monopodial branches and 25–26 sympodia. Each node of the sympodium bears 2–3 fruiting branches (sympodial branches). Each sympodium consists of 4–12 nodes, with each node bearing one determinate and one indeterminate fruiting branch. The internode length ranges from 15 to 30 cm on sympodial branches, while the internode length on the main stem is 5–10 cm. Leaves are medium-sized (9.5 × 10.0 cm), green, 7-lobed, with broad lobes, and very strongly pubescent. Each leaf has one triangular, colorless nectary. The petiole is 7.5 cm long and exhibits a strong anthocyanin blush. There are two persistent, lanceolate stipules. The flower is medium-sized (6.5–7.0 cm), bell-shaped, and opens to a medium width. The pedicel is 4.5–5.0 cm long, has a weak anthocyanin blush, and is covered with small gossypol glands. There are three green, cordate bracts (epicalyx) with 7–8 weakly serrated teeth. The bracts lack external nectaries, but have three internal, round, colorless nectaries. The cotton boll is entire, light green, and covered with gossypol glands. The corolla is medium-sized (5.5 × 6.0 cm), yellow, and has a large, prominent anthocyanin spot at the base. The staminal column is conical, 4.0–4.5 cm high, with anthers arranged at medium density. The anthers and filaments are dark yellow, while the filaments themselves are purple. The pistil has three stigmas, which protrude 1.0–1.2 cm beyond the anthers. The fiber is 14.0–16.0 mm long, light brown-colored, smooth, soft, and with high strength (Figure 2).

**Figure 3:**
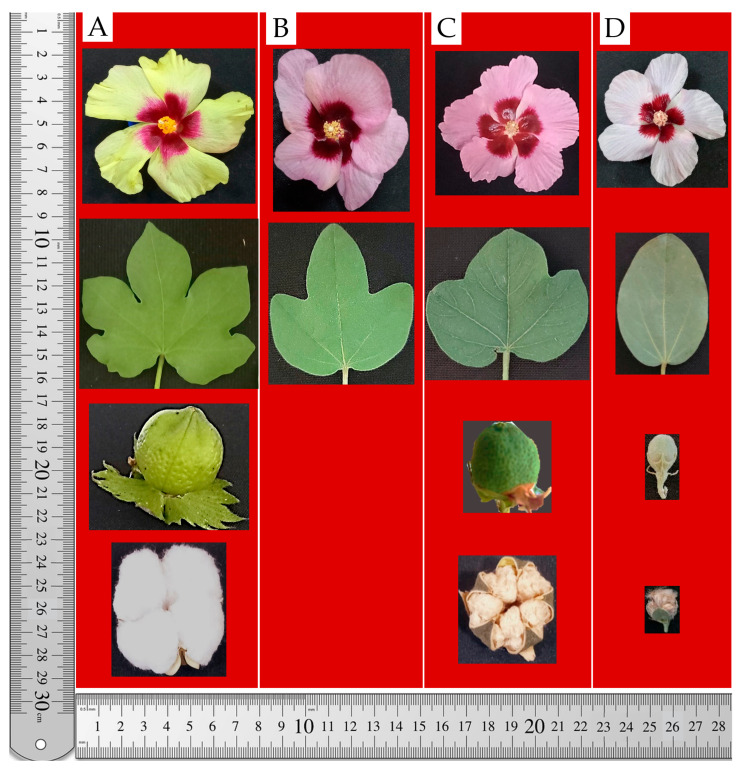
Morphological characteristics of cotton genotypes. (**A**) *G. herbaceum* subsp*. euherbaceum* cv. А-256—(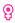) diploid maternal genotype; (**B**) diploid first generation—(F_1_) *G. herbaceum* subsp*. euherbaceum* cv. А-256 *× G. nelsonii*; (**C**) allotetraploid hybrid progeny of the first generation—(F_1_C) *G. herbaceum* subsp*. euherbaceum* cv. А-256 *× G. nelsonii*; (**D**) *G. nelsonii*—(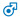) diploid paternal genotype.

The F_1_C *G. herbaceum* subsp. *euherbaceum* (cv. А-256) × *G. nelsonii* hybrid has an erect, branched stem with medium-density leaf arrangement (Figure 3). The main stem height ranges from 240.0 to 250.0 cm and is light green and strongly pubescent. The total number of nodes is up to 44. Branching is sympodial, with the first sympodial fruiting branch forming at node 14. There are 2–3 monopodial branches and 38–40 sympodia. Leaves are large (20.5 × 18.5 cm), light green, palmate, 3–5 lobed, with rounded lobes, and strongly pubescent. Each leaf has one colorless, lanceolate nectary. The petiole is 12–15 cm long, green, with a weak anthocyanin blush at the base and apex. There are two persistent, lanceolate stipules. The flower is medium-sized (6.0 cm), bell-shaped, and opens to a medium width. The pedicel is short (0.5–1.0 cm), covered with small gossypol glands, and has a weak anthocyanin blush. There are three light green, lanceolate bracts (epicalyx) with 3–6 weakly serrated teeth that are 0.1–0.3 cm long, each covered with small gossypol glands and a weak anthocyanin blush. The bracts have three large, round, colorless external nectaries and three internal nectaries that are also colorless and covered with small gossypol glands. The cotton boll has five lobes, is light green, with teeth that are 0.5 cm long, and has a weak anthocyanin blush. The corolla (with 6 petals, 1 of which is small and underdeveloped) is medium-sized (6.0 × 6.5 cm), light purple or pink, with a strong anthocyanin spot at the base. The staminal column is conical, has a moderate anthocyanin blush, is covered with very small, sparse gossypol glands, and is 2.5 cm high. The anthers are dark, and densely arranged. The anthers are light pink, the filaments are light yellow, and the filament itself is dark red. The pistil has four stigmas, with the stigma being level with the staminal column and not protruding. The boll is small, spherical, rough, and sparsely covered with hairs. It has 4–5 locules. The fiber is 17.0–19.0 mm long, light cream-colored, smooth, soft, and with high strength.

#### 2.2.3. Inheritance and Improvement of Fiber Traits in Synthetic Hybrids

Fiber length is one of the most important economic traits in cotton, as it directly affects spinning performance and the commercial value of the raw material. In this study, fiber length was assessed in the F_1_C allotetraploid hybrids and their diploid parental genotypes. The results revealed significant differences in fiber length across the genotypes.

*G. herbaceum* subsp. *euherbaceum* (cv. A-256) produced relatively long fibers measuring 26–28 mm, while *G. nelsonii* had very short fibers averaging only 7.0–8.0 mm. The F_1_C hybrid obtained from the A-256 × *G. nelsonii* cross exhibited an intermediate fiber length of 17.0–19.0 mm. This suggests a possible heterotic effect or an additive inheritance pattern that contributed to fiber elongation in the F_1_C generation.

**Figure 4:**
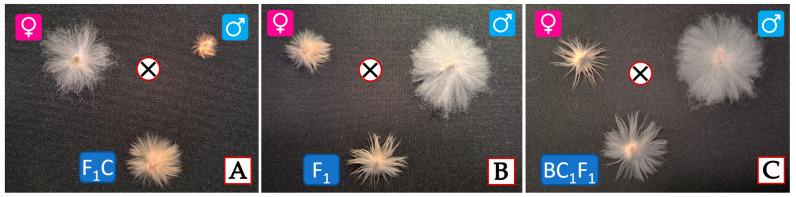
Fiber development in parental and hybrid genotypes: (**A**) 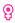
—*G. herbaceum* subsp. *frutescens*, 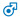
—diploid *G. nelsonii*, F_1_C—synthetic hybrid of *G. herbaceum* subsp*. frutescens* × *G. nelsonii* combination; (**B**) 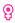
—F_1_C synthetic hybrid of *G. herbaceum* subsp*. frutescens* × *G. nelsonii* combination, 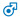
—*G. hirsutum* cv. Ravnak-1, F_1_—first generation hybrid of [(*G. herbaceum* subsp*. frutescens* × *G. nelsonii*) × *G. hirsutum* cv. Ravnak-1]; (**C**) 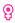
—F_1_ hybrid of [(*G. herbaceum* subsp*. frutescens* × *G. nelsonii*) × *G. hirsutum* cv. Ravnak-1], 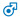
—*G. hirsutum* cv. Ravnak-1, BC_1_F_1_—backcross hybrid (BC_1_F_1_) [(*G. herbaceum* subsp*. frutescens* × *G. nelsonii*) × *G. hirsutum* cv. Ravnak-1]. **Note:** The 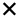 symbol denotes a hybridization (cross) between the respective parental genotypes.

Similarly, the F_1_C hybrid derived from the subsp. *frutescens* × *G. nelsonii* cross also showed longer fiber than either parent, averaging 14.0–16.0 mm (Figure 4A). These results showed that *G. nelsonii* genomic components integrated into the *G. herbaceum* background can enhance fiber development and suggest that fiber length is likely a polygenic trait with high plasticity in interspecific allotetraploids.

All the F_1_C genotypes exhibited a low phenotypic coefficient of variation (V < 5%) for fiber length, indicating a stable expression of the trait. Overall, these findings highlight the importance of synthetic allotetraploids as a valuable genetic bridge for introducing new fiber quality traits into cultivated cotton germplasm.

Further hybridization between the F_1_C allotetraploids and a local moderate-fiber quality variety (*G. hirsutum* cv. Ravnak-1) resulted in the development of complex F_1_ hybrids [(A-256 × *G. nelsonii*) × Ravnak-1] and [(*frutescens* × *G. nelsonii*) × Ravnak-1] which produced fibers measuring 20.0–22.0 mm in length (Figure 4B).

These complex F_1_ hybrids were then backcrossed with the local *G. hirsutum* variety. As a result, we obtained backcross (BC_1_F_1_) hybrids with improved fiber characteristics: fine, glossy, strong fibers with an average length of 25.0–26.0 mm (Figure 4C).

These findings showed that fiber length is a highly variable and heritable trait in synthetic allotetraploid hybrids derived from *G. herbaceum* and *G. nelsonii*. The observed improvements in fiber length and quality through reciprocal hybridization and backcrossing with *G. hirsutum* suggest the potential of these wild-derived synthetic cottons for enhancing cultivated cotton germplasm. These results support the feasibility of utilizing interspecific and complex hybrids as a genetic bridge to introgress desirable fiber traits into elite cotton cultivars.

### 2.3. Wild Species-Derived Insect Pest Resistance in Synthetic Allotetraploids

#### 2.3.1. Resistance to Cotton Aphids (*Aphis gossypii* Glover.)

The study evaluated the resistance of parental lines, F_1_, F_1_C, and complex F_1_ hybrids to cotton aphids (Table 4). The wild diploid species *G. nelsonii* exhibited the highest resistance among parental lines, with an infestation rate of 6.01% and a variability amplitude of 4.7–7.7%.

In contrast, cv. Ravnak-1 (subsp. *euhirsutum*) was highly susceptible, showing 22.19% infestation and a low coefficient of variation (4.08%). The F_1_C hybrids, particularly subsp. *frutescens* × *G. nelsonii* (5.16%) and its reciprocal (5.56%), demonstrated strong resistance, with variability ranges of 4.3–6.0% and 4.3–6.7%, respectively. Complex F_1_ hybrids involving Ravnak-1 exhibited moderate resistance (6.56–8.0%), indicating intermediate tolerance to this insect.

#### 2.3.2. Resistance to Whiteflies (Aleyrodidae)

**Figure 5:**
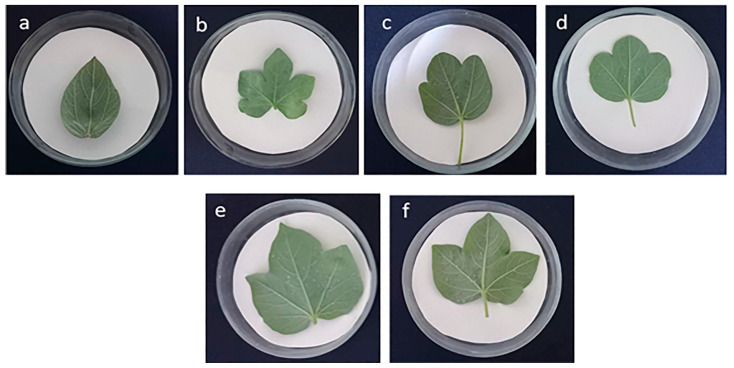
(**a**) *G.nelsonii*, (**b**) subsp. *frutescens*, (**c**) F_1_ (subsp. *frutescens* × *G.nelsonii*), (**d**) F_1_C (subsp. *frutescens* × *G.nelsonii*), (**e**) *G. hirsutum* Ravnak-1, (**f**) F_1_ (subsp. *frutescens* × *G. nelsonii*) × Ravnak-1.

The resistance to whiteflies (Aleyrodidae) was also assessed (Table 4, Figure 5). *G. nelsonii* displayed the highest resistance among the parental lines (6.3% infestation, CV 9.83%), while Ravnak-1 was highly susceptible (23.9% infestation, CV 5.14%).

The F_1_C hybrids subsp. *frutescens* × *G. nelsonii* (5.4%) and its reciprocal (5.8%) exhibited strong resistance, with variability amplitudes of 4.4–6.5% and 4.9–6.6%, respectively. Complex F_1_ hybrids with Ravnak-1 displayed moderate resistance (7.5–10.6%), with higher variability (V = 9.8–11.67%).

These results demonstrated the potential of *G. nelsonii* and its hybrid derivatives in breeding programs aimed at enhancing insect pest resistance in cotton.

### 2.4. Cytogenetic Analysis of Interspecific Hybrids

#### 2.4.1. Cytogenetic Screening of Polyploid Hybrids

**Figure 6:**
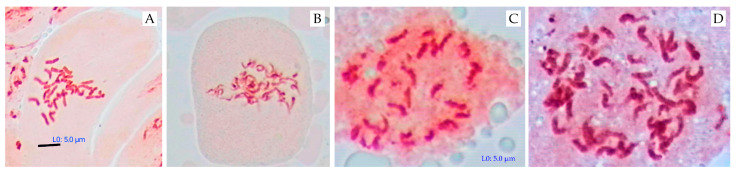
Metaphase chromosomes in *Gossypium* species and hybrids. (**A**) *G. herbaceum* (2n = 26); (**B**) *G. nelsonii* (2n = 26); (**C**) F_1_ (*G. herbaceum* × *G. nelsonii*) (2n = 26); (**D**) F_1_C (*G. herbaceum* × *G. nelsonii*) (2n = 52). Scale bar = 5.0 µm.

Cytogenetic analysis of mitosis was performed on the F_1_ hybrid *G. herbaceum* × *G. nelsonii*, its parental forms (*G. herbaceum* and *G. nelsonii*), and colchicine-treated polyploid allotetraploids. As expected, the diploid parental species, *G. herbaceum* (Figure 6A) and *G. nelsonii* (Figure 6B), as well as their untreated F_1_ hybrid (Figure 6C), exhibited a chromosome complement of 2n = 26. In contrast, the colchicine-treated (A_1_ × G) hybrid displayed a doubled chromosome set of 2n = 52 (Figure 6D), confirming the successful induction of polyploidy. These results indicate that the chromosome doubling in the F_1_ hybrid led to the formation of an allotetraploid.

The interspecific F_1_ hybrids were sterile due to a lack of flower development, while the colchicine-treated polyploid hybrids were fertile and produced normal offspring. Cytological analysis of the F_1_ hybrid plants revealed 13 bivalents in the pollen mother cells during metaphase I (MI) of meiosis, indicating normal meiotic progression.

In interspecific hybrids, incompatibility between the nucleus and the cytoplasm can disrupt the development of generative tissues. This disruption often manifests as abnormalities during mitosis and meiosis, particularly affecting chromosome conjugation during sporogenesis. Consequently, an incorrect distribution of genetic information can occur, leading to reduced viability or sterility in hybrid plants. Tetrad analysis is a valuable tool for identifying these meiotic irregularities.

Meiosis typically culminates in the formation of four haploid microspores or megaspores, collectively known as a tetrad. In a normal tetrad, the segregation of any pair of allelic genes results in a 2:2 ratio. However, deviations from this expected pattern can occur and are detectable through tetrad analysis [17].

In this study, tetrad analysis of the interspecific hybrids revealed a range of meiotic abnormalities. While some pollen mother cells (PMCs) exhibited normal meiotic behavior, including the formation of normal dyads, tetrads, and ultimately, pollen grains, others displayed various abnormalities. These included unequal separation of dyads, formation of triads due to asynchronous division, and the presence of polyads (pentads, hexads, heptads, etc.) during telophase II (Figure S2, Table 5). For instance, out of the 500 analyzed spore products, 104 (20.80%) were dyads, 44 (8.80%) were triads, 197 (39.40%) were tetrads, and 155 (31.00%) were other polyads. Among the 197 tetrads, 120 (60.91%) were normal, while 77 (39.09%) contained micronuclei, indicative of chromosomal aberrations. These abnormal tetrads likely contributed to the observed pollen sterility, as evidenced by the presence of malformed pollen grains, including large, oval-shaped grains and small, immature grains. These meiotic irregularities are likely attributable to a slight asynchrony in chromatid separation during anaphase I of hybrid meiosis. The presence of micronuclei in a significant proportion of tetrads suggests chromosomal instability and likely contributes to the observed sterility in the F_1_ hybrids.

In the F_1_ *G. herbaceum* subsp. *frutescens* × *G. nelsonii* hybrid, the proportion of micronucleated spores was 20.3 ± 1.7%, the proportion of polyads was 5.5 ± 0.9%, and the meiotic index was 57.8 ± 2.1%.

In the F_1_ *G. herbaceum* subsp. *euherbaceum* (cv. А-256) × *G. nelsonii* hybrid, the proportion of micronucleated spores was 6.9 ± 0.8%, the proportion of polyads was 2.5 ± 0.5%, and the meiotic index was 78.6 ± 1.3%.

In the F_1_C allotetraploid hybrids (*G. herbaceum* subsp. *frutescens* × *G. nelsonii* and *G. herbaceum* subsp. *euherbaceum* (cv. А-256) × *G. nelsonii*), the meiotic indices were similar. Specifically, in the F_1_C *G. herbaceum* subsp. *frutescens* × *G. nelsonii* allotetraploid hybrid, the proportion of micronucleated tetrads was 37.6 ± 2.5%, the proportion of polyads was 1.8 ± 0.7%, and the meiotic index was 46.8 ± 2.6%.

In the F_1_C *G. herbaceum* subsp. *euherbaceum* (cv. А-256) × *G. nelsonii* allotetraploid hybrid, these figures were 36.7 ± 2.5% for micronucleated tetrads, 1.9 ± 0.7% for polyads, and 47.1 ± 1.9% for the meiotic index.

#### 2.4.2. Pollen Fertility

Plant pollen quality and fertility are crucial for successful fertilization and the production of viable offspring. Pollen fertility can also be used to assess genetic differentiation and phylogenetic relationships between species [2,17]. Pollen development (microsporogenesis) and fertilization are sensitive to various abiotic stresses, including adverse weather conditions, nutrient deficiencies, temperature extremes, chemical exposure, and radioactive pollution [25].

First-generation hybrids resulting from crosses between distantly related wild species of the A_1_ and G genomes exhibited very low pollen viability, leading to sterility or partial sterility.

The pollen viability of the F_1_ hybrids *G. herbaceum* subsp. *frutescens* × *G. nelsonii* (4.56 ± 0.35%), *G. nelsonii* × *G. herbaceum* subsp. *frutescens* (4.72 ± 0.33%), *G. herbaceum* subsp. *euherbaceum* (cv. А-256) × *G. australe* (4.82 ± 0.67%), and *G. herbaceum* subsp. *euherbaceum* (cv. А-256) × *G. bickii* (4.73 ± 0.55%) was found to be similarly low. In contrast, the pollen viability of the F_1_ hybrids *G. nelsonii* × *G. herbaceum* subsp. *pseudoarboreum* f. *harga* and *G. herbaceum* subsp. *euherbaceum* (cv. А-256) × *G. nelsonii* was slightly higher, at 7.29 ± 0.41% and 7.07 ± 0.91%, respectively. Among the F_1_ hybrids, *G. herbaceum* subsp. *pseudoarboreum* f. *harga* × *G. nelsonii* exhibited the highest pollen viability (8.79 ± 1.57%). The lowest pollen viability was observed in the F_1_ *G. herbaceum* subsp. *pseudoarboreum* × *G. australe* hybrid (2.2 ± 0.17%). These low pollen viability percentages are likely a major contributing factor to the observed sterility in these interspecific hybrids. Cytological studies revealed that fertility was restored in the F_1_C allotetraploid hybrids. Specifically, the F_1_C *G. herbaceum* subsp. *frutescens* × *G. nelsonii* allotetraploid hybrid exhibited a pollen viability of 47.2 ± 1.04%, while the F_1_C *G. herbaceum* subsp. *euherbaceum* (cv. А-256) × *G. nelsonii* hybrid showed an increased pollen viability of 54.1 ± 1.04% (Table 6, Figure S3).

#### 2.4.3. Molecular Genetic and Phylogenetic Analysis of the Intraspecific Hybrids

**Figure 7:**
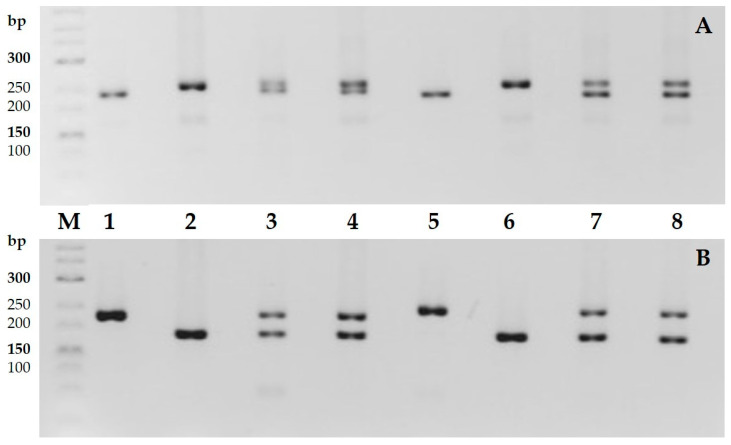
Genetic polymorphisms of the cotton samples using SSR markers. (**A**) BNL1122 marker; (**B**) BNL1679 marker. 1—*G. herbaceum* subsp. *frutescens*; 2—*G. nelsonii*; 3—F_1_ (*G. herbaceum* subsp. *frutescens* × *G. nelsonii*); 4—F_1_C (*G. herbaceum* subsp. *frutescens* × *G. nelsonii*); 5—*G. herbaceum* subsp. *euherbaceum* (cv. А-256); 6—*G. nelsonii*; 7—F_1_ (*G. herbaceum* subsp. *euherbaceum* (cv. А-256) × *G. nelsonii*); 8—F_1_C (*G. herbaceum* subsp. *euherbaceum* (cv. А-256) × *G. nelsonii*).

To determine the genetic polymorphism between the parental forms, PCR analysis was performed using 192 SSR markers. Of these, 74 markers showed polymorphism among the cotton samples, while 92 were monomorphic, and 26 failed to amplify any of the cotton genotypes (Figure 7).

In this part of the study, to confirm the hybridity of the F_1_ hybrids, polymerase chain reaction (PCR) analysis was performed using four polymorphic SSR markers: BNL1122, BNL1679, NAU3093, and JESPR156. These markers were selected based on their high polymorphism in the parental forms. PCR screening results confirmed that the F_1_ hybrids contained alleles inherited from both parental species, *G. herbaceum* subsp. *euherbaceum* (A_1_) and *G. nelsonii* (G_3_) (Figure 7).

**Figure 8:**
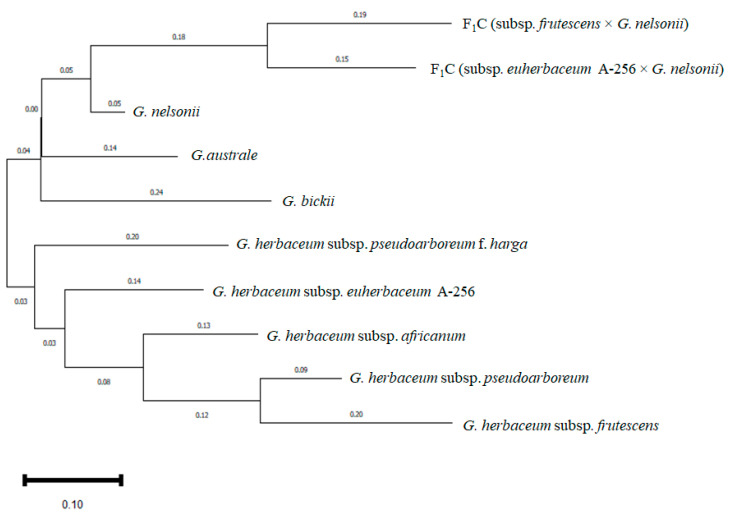
Phylogenetic tree depicting the genetic relationships among the *Gossypium* species and subspecies, including F_1_C samples derived from interspecific crosses (subsp. *frutescens* × *G. nelsonii* and subsp. *euherbaceum* A-256 × *G. nelsonii*), based on 74 polymorphic SSR markers. Both F_1_C samples and their respective parental genotypes were analyzed together, highlighting their phylogenetic proximity and divergence. The tree was constructed using the neighbor-joining method in PAUP 4.0 (Phylogenetic Analysis Using Parsimony and other methods). Branch lengths are proportional to genetic distance, as indicated by the scale bar (0.10).

The genetic relationships among the studied *Gossypium* species, including *G. herbaceum* subspecies, Australian wild species, and two interspecific F_1_C hybrids, were evaluated through phylogenetic analysis based on 74 polymorphic SSR markers. The phylogenetic tree constructed using the neighbor-joining method in PAUP 4.0 (Phylogenetic Analysis Using Parsimony and other methods) is presented in Figure 8.

The tree shows two major clusters. The first cluster includes all subspecies of *G. herbaceum* and the interspecific F_1_C hybrid (subsp. *euherbaceum* A-256 × *G. nelsonii*), while the second cluster comprises the three Australian wild species—*G. bickii*, *G. australe*, and *G. nelsonii*—along with the interspecific F_1_C hybrid (subsp. *frutescens* × *G. nelsonii*).

Within the *G. herbaceum* cluster, subsp. *euherbaceum* (cv. A-256) and subsp. *pseudoarboreum* f. *harga* displayed the closest relationship, forming a distinct subcluster with a genetic distance of 0.14. Subsp. *frutescens*, subsp. *pseudoarboreum*, and subsp. *africanum* formed another subcluster.

However, contrary to the initial expectations, both F_1_C samples—subsp. *frutescens* × *G. nelsonii* and subsp. *euherbaceum* A-256 × *G. nelsonii*—were not positioned near their maternal genotypes. Instead, they were placed closer to the paternal genotype, *G. nelsonii*, in the phylogenetic tree, indicating a dominant inheritance of paternal traits. Our study also observed that in both direct and reciprocal crosses between *G. nelsonii* and *G. herbaceum*, the morphological and biological traits of *G. nelsonii* were dominantly inherited regardless of whether it served as the male or female parent.

In addition, the observed placement of F_1_C hybrids might be attributed to the marker bias toward *G. nelsonii* alleles used in constructing the tree, or to asymmetrical interactions between parental genomes in early-generation hybrids.

In the second cluster, *G. nelsonii* exhibited greater genetic similarity to *G. australe* (0.07) than to *G. bickii* (0.21). The F_1_C hybrid derived from subsp. *frutescens* × *G. nelsonii* was also positioned close to *G. nelsonii*, consistent with its paternal origin. The branch lengths, representing genetic distances (scale bar = 0.10), highlight the substantial divergence between the *G. herbaceum* group and the Australian species.

Importantly, the interspecific F_1_C hybrid between subsp. *euherbaceum* A-256 and *G. nelsonii* showed a pollen viability of 54.1 ± 1.04% (Table 6, Figure S3), indicating a certain degree of genomic compatibility between the two parental lines despite their phylogenetic divergence.

## 3. Discussion

The domestication of *Gossypium hirsutum* has profoundly transformed wild cotton species into the modern cultivars cultivated today [26]. This extensive process of artificial selection, spanning thousands of years, has led to significant morphological and physiological changes, including longer and stronger fibers, improved yield, and adaptation to diverse environments. However, this transformation has also resulted in a marked reduction in genetic diversity compared to their wild progenitors [1,7,9]. Studies have shown that modern upland cotton cultivars exhibit decreased genetic variation, particularly in the genes associated with stress responses, making them more susceptible to biotic and abiotic stresses [13,27,28,29].

In our studies, we successfully obtained hybrids from representatives of the wild Australian species *G. nelsonii* Fryx and the Afro-Asian species *G. herbaceum* to introduce valuable traits into the cultivated species *G. hirsutum* L. Similar studies have successfully developed synthetic allotetraploid cotton genotypes by interspecific hybridization between distantly related species. For instance, Yin et al. (2020) created a novel allotetraploid genotype (A_1_A_1_G_3_G_3_) through hybridization between *Gossypium herbaceum* and *G. nelsonii*, followed by chromosome doubling using colchicine treatment [18]. This synthetic allotetraploid exhibits enhanced disease resistance and serves as a valuable genetic resource for cotton breeding programs.

In another study, Liu et al. (2015) developed a synthetic allotetraploid (A_1_A_1_G_2_G_2_) by crossing *G. herbaceum* with *G. australe* [28]. The resulting hybrid combined favorable traits from both diploid species, such as drought tolerance and resistance to pests and diseases, and demonstrated potential for transferring these traits into cultivated upland cotton (*G. hirsutum*) through bridge crossing.

Khidirov et al. [17] succeeded in obtaining triploid hybrids by synthetic polyploidy by crossing *G. herbaceum* (A_1_) and *G. mustelinum* Miers ex Watt (AD_4_). Due to the complexity of genetic exchange between chromosomes of distant species such as *G. australe* (2n = 26, GG) and *G. hirsutum* (2n = 52, AADD), the probability of transferring the desired traits to cultivated varieties is considered very low. To somewhat facilitate the introgression of such unique genes into cultivated varieties, monosomic alien chromosome addition lines (MAALs) were created using *G. hirsutum* and *G. australe* [29]. In addition, translocation lines were obtained by inducing chromosome introgression and translocation from *Gossypium australe* to *Gossypium hirsutum* by Wang et al. [30].

In this study, we also observed an interesting inheritance pattern of fiber pigmentation in hybrids involving *G. nelsonii*, *G. herbaceum*, and *G. hirsutum*. While *G. nelsonii* fibers were brown and *G. herbaceum* subsp. *frutescens* fibers were white, the F_1_ hybrids between them showed a diluted brown color. However, this pigmentation was further diminished or nearly lost in the BC_1_F_1_ generation involving *G. hirsutum*, suggesting suppression of brown coloration. Based on this observation, we hypothesize that the brown fiber trait from *G. nelsonii* is subject to dominant epistatic suppression by genomic elements of *G. hirsutum*. This implies that *G. hirsutum* may harbor regulatory loci that inhibit the expression of pigmentation genes inherited from wild species. Additional molecular studies, including gene expression analysis, are required to validate this hypothesis and evaluate the potential of directing these regulatory elements for breeding purposes.

The restoration of pollen fertility in synthetically developed allotetraploids opens new possibilities for their utilization in cotton genetic improvement programs [17,18,28]. In our study, several lines derived from the F_1_C and subsequent backcross generations exhibited improved fiber traits, particularly enhanced fiber length and strength. Moreover, these lines also demonstrated promising yield potential under field conditions. These observations suggest that the introgressed traits are, at least partially, heritable and may be retained in advanced backcross progenies, potentially contributing to the recovery and improvement of the recipient genome.

At the same time, favorable genes are currently being introgressed into *G. hirsutum* cultivars using the marker-assisted backcrossing (MABC) technology. The resulting backcross progenies have been advanced up to the sixth generation and are being proposed for agronomic testing as potential new cultivars.

## 4. Materials and Methods

### 4.1. Plant Materials

Diploid (2n = 2x = 26) cotton species, including *Gossypium herbaceum* subspecies (A_1_A_1_), *G. bickii* (G_1_G_1_), *G. australe* (G_2_G_2_), *G. nelsonii* (G_3_G_3_), and the two *G. hirsutum* elite cultivars Ravnak-1, Ravnak-2, and Baraka (commercial varieties from Uzbekistan), were used in our experiments (Table S1). The materials were obtained from the cotton germplasm collection at the Institute of Genetics and Plant Experimental Biology, Academy of Sciences of the Republic of Uzbekistan.

### 4.2. Hybridization and Polyploidization

Hybridization processes of cotton genotypes were carried out at the experimental field of the Laboratory of Experimental Polyploidy and Phylogeny (41°20′08.5″ N 69°20′25.2″ E), Tashkent, Uzbekistan, in 2022–2024. The hybridization process involved several key steps. First, anthers were removed from the maternal plant’s flowers before they matured to prevent self-pollination. Then, the flowers were covered with paper bags to protect them from unwanted pollination. Next, pollen from the paternal plant was applied to the maternal plant’s stigma, typically between 9:00 AM and 12:00 PM. After pollination, the flowers were re-covered with bags and labeled. Finally, once the bolls matured, they were harvested, and the seeds were extracted and planted in pots.

The Australian wild diploid (2n = 2x = 26) species (*G. bickii*, *G. australe*, and *G. nelsonii*) were hybridized with the Afro-Asian diploid (2n = 2x = 26) *G. herbaceum* subspecies (subsp. *africanum*, subsp. *pseudoarboreum*, subsp. *pseudoarboreum* f. *harga*, subsp. *frutescens*, and subsp. *euherbaceum* cv. А-256). All hybrid combinations were obtained by direct and reciprocal crossing.

To obtain allotetraploid polyploids from diploid hybrids, 0.2% colchicine was applied for three different durations: 18, 20, and 24 h. All treatments were conducted in darkness at room temperature (22 °C), with applications ceasing when shoots reached approximately 1 cm in length. The 18 h treatment failed to induce polyploidy, while the 24 h treatment proved lethal. Success was achieved only with the 20 h treatment, consistent with the findings of [21]. Consequently, fertile F_1_C (C for colchicine) synthetic allotetraploid (2n = 4x = 52) hybrid genotypes were obtained only from the subsp. *euherbaceum* (cv. А-256) × *G. nelsonii* and subsp. *frutescens* × *G. nelsonii* crosses. Then, the synthetic allotetraploid hybrids were hybridized with *G. hirsutum* subsp. *euhirsutum* cultivars Ravnak-1, Ravnak-2, and Baraka.

### 4.3. Phenological Observations

Field evaluations were conducted during the 2022–2023 growing seasons to assess phenotypic traits in the *G. herbaceum* subsp. *euherbaceum*, (cv. А-256), subsp. *frutescens*, *G. nelsonii*, and *G. hirsutum* subsp. *euhirsutum* cultivars Ravnak-1, Ravnak-2, and Baraka and their derived F_1_C allotetraploids. Morphobiological characteristics, such as vegetative growth period, plant height, stem color and hairiness, leaf shape, flower structure and color, number of bolls per plant, boll weight and shape, number of seeds per boll, 1000-seed weight, fiber length, fiber strength, and pollen fertility/sterility, were observed.

### 4.4. Insect Pest Resistance Evaluation

#### 4.4.1. Experimental Design

The insect pest resistance evaluation was conducted as follows:

Test samples: parental lines (*G. herbaceum* subspecies and *G. nelsonii*), F_1_ hybrids, F_1_C allotetraploids, and complex F_1_ hybrids (F_1_ × *G. hirsutum* cv. Ravnak-1), with five biological replicates per genotype.

Control samples: commercial *G. hirsutum* cv. Ravnak-1 as the susceptible control.

Growth conditions: plants were maintained in controlled greenhouse chambers at 25 ± 2 °C, 60% relative humidity, with 16 h light.

#### 4.4.2. Insect Infestation Assays

Aphid bioassay:

1. Aphid colonies were maintained on susceptible cotton plants under greenhouse conditions.
2. For experiments, 10 aphids were transferred to each test plant using a fine brush.
3. Infestation severity was assessed every 48 h for 21 days using a standardized 0–5 scale:

0—no visible damage

1—slight leaf curling

3—moderate curling with some stunting

5—severe curling/necrosis and growth arrest

4. Data from 10 replicates per genotype were used to calculate mean infestation percentages (Table 4).

Whitefly bioassay:

1. Whitefly adults were collected from infected field plants and maintained in insectaries.
2. Test plants were infested with 15 adult whiteflies per plant, confined in mesh cages.
3. Damage assessment included:
  - Percentage of the chlorotic leaf area;
  - Nymph density per cm^2^ of leaf surface;
  - Overall plant vigor score (1–5 scale).
4. The results were statistically analyzed as described in Section 4.7 and presented in Table 4.

Quality control measures:

- All the experiments included a randomized complete block design;
- Insect populations were standardized by age and size before inoculation;
- Environmental conditions were monitored continuously using data loggers;
- Control genotype (cv. Ravnak-1) was included in each experimental run.

This standardized protocol enabled reproducible evaluation of pest resistance among different cotton genotypes while minimizing environmental variability. The quantitative scoring system allowed for direct comparison between wild relatives, synthetic hybrids, and cultivated varieties.

### 4.5. Cytological Analysis

Cytogenetic studies were conducted following methodologies established in previous research [17]. The analysis encompassed metaphase-1 (M1) of meiosis in pollen mother cells (PMCs) and the assessment of spore and pollen grain viability. For this purpose, plant buds were fixed in a 3:7 ethanol–acetic acid mixture and selected for microscopic observation of meiosis and tetrad analysis. Preparations were made using aceto-carmine stain. The meiotic index was determined by calculating the proportion of normal tetrads out of the total number of spores, as described in previous studies [2].

To assess pollen viability, fully opened flowers from the parental lines, F_1_, and F_1_C hybrid generations were collected at approximately 10:00 AM, and temporary preparations were made. Aceto-carmine stain was used to stain pollen grains, and the preparations were incubated at +4 °C for 24 h to enhance staining intensity. Analyses were performed using a Leica CME microscope (Leica Microsystems, Wetzlar, Germany) equipped with a Leica EC3 camera (Leica Microsystems, Germany), an XSP-500SM trinocular microscope (Ningbo Shinea IMP. & EXP. Co. Ltd., Ningbo, China), and a UM039 portable digital microscope (Terrific, Beijing, China).

For tetrad analysis, flower buds measuring 2–4 mm were collected between 9:00 and 10:00 AM and fixed in a 3:7 ethanol–acetic acid mixture. Temporary preparations were made using aceto-carmine stain and observed under a light microscope. The materials were sorted to isolate the desired meiotic stages. The meiotic index, defined as the proportion of normal tetrads to the total number of sporads, was calculated.

### 4.6. Genomic DNA Isolation and SSR Marker Analysis

Genomic DNA was extracted from the fresh leaf tissues of cotton seedlings. The tissues were then frozen in liquid nitrogen and ground to a fine powder. DNA samples were then isolated using a modified cetyltrimethylammonium bromide (CTAB) method [31]. DNA purity and concentration were assessed using a NanoDrop 2000 spectrophotometer (Thermo Fisher Scientific, Waltham, MA, USA) with absorbance measurements taken at 260 nm and 280 nm. DNA samples were diluted to a working concentration of 25 ng/µL for subsequent analyses.

To assess genetic polymorphism between the parental lines, polymerase chain reaction (PCR) analysis was performed using 192 microsatellite markers (DPL, Gh, HAU, JESPR, and BNL collections) associated with economically important traits of cotton, including fiber quality, yield, and disease resistance. Of these, 156 markers revealed polymorphism between the parental genotypes. These polymorphic markers were then used to validate the F_1_ hybrid status. PCR products were visualized using gel electrophoresis, and genotype data were analyzed using Gel Analyzer v.19.1 software. The resulting genotypic data were organized in Microsoft Excel and used to construct a phylogenetic tree using NCSS v.12.0.2 software.

### 4.7. Statistical Analysis

Statistical analyses were performed using the methods described by Maxwell and Delaney [32] as a guideline. For each parameter, the mean and standard deviation were calculated from ten replicates (n = 10). The standard deviation (σ) was determined using the following formula:

$$\sigma =\sqrt{\frac{\sum {\left(X_{i}-\bar{X}\right)}^{2}}{n-1}}$$

where *X_i_* represents each individual measurement, $\bar{X}$ represents the arithmetic mean of the measurements, and *n* represents the number of measurements.

The standard error of the mean ($\sigma_{\bar{X}}$) was then calculated as follows:

$$\sigma_{\bar{X}}=\frac{\sigma }{\sqrt{n-1}}$$

To determine the 95% confidence interval, the Student’s *t*-coefficient ($t_{c}$) for n − 1 degrees of freedom was used. The confidence interval (Δ) was calculated as follows:

$$\Delta =t_{c}\sigma_{\bar{X}}$$

The relative error (S) of the measurements, expressed as a percentage, was calculated using the following formula:

$$S=\frac{100\sigma_{\bar{X}}t_{c}}{\bar{X}}$$

Finally, the coefficient of variation (V), also expressed as a percentage, was determined using the following equation:

$$V=\frac{100\sigma_{\bar{X}}}{X}$$

The results of the phylogenetic analysis were visualized using MEGA v.11.0.13 software [33].

### 4.8. Phylogenetic Analysis

To investigate the genetic relationships among the cotton species and their interspecific allopolyploid hybrids, a phylogenetic analysis was performed. 74 polymorphic SSR markers (Table S2) were used to assess genetic distances between the accessions.

These distances were then used to construct a phylogenetic tree employing the neighbor-joining algorithm within the PAUP v.4.0 software package (Phylogenetic Analysis Using Parsimony and other methods) [34]. This analysis provided insights into the evolutionary relationships and genetic variation of the studied cotton accessions.

## 5. Conclusions

In this study, we demonstrated the feasibility of enriching the genetic base of cultivated cotton (*G. hirsutum* L.) by utilizing synthetic allotetraploids derived from interspecific hybridization between Afro-Asian *G. herbaceum* and Australian wild cotton species *G. nelsonii*. The successful generation of fertile allotetraploid hybrids opens new avenues for introgression of valuable traits from wild cotton species into cultivated varieties.

Cytogenetic analysis confirmed the successful induction of polyploidy in the F_1_ hybrids, leading to the formation of fertile allotetraploids. Molecular marker analysis using SSRs revealed genetic variation among the parental lines and validated the hybrid nature of the progeny. The synthetically generated allotetraploids exhibited restored pollen viability and represent a valuable resource for introgression traits such as disease resistance, drought tolerance, and fiber quality from wild *Gossypium* species into cultivated upland cotton.

Moreover, inheritance analysis revealed that brown fiber pigmentation from *G. nelsonii* appears to be suppressed in backcross generations involving *G. hirsutum*, suggesting the possible presence of regulatory loci in *G. hirsutum* that may control pigmentation. This insight provides a promising basis for future gene expression studies aimed at understanding the epistatic interactions between cultivated and wild cotton genomes.

Further research is needed to explore the genetic potential of these allotetraploids and develop advanced breeding lines with improved agronomic performance and enhanced resistance to biotic and abiotic stresses. By harnessing the genetic variation of wild cotton species, we can contribute to the development of sustainable and resilient cotton cultivars for the future.

## Figures and Tables

**Table 1:** The number of bolls per parental plant and the fertility of cotton seeds.

No.	Plant Samples	Number of Bolls		Number of Seeds per Boll			Complete Seeds per Boll, %			
		Per Plant	Analyzed	Total	Complete	Empty	$\bar{x}\pm S\bar{x}$	Range	S	V %
*G. herbaceum* subspecies and forms										
1.	*G. herbaceum* subsp. *africanum*	28	10	15.0	13.0	2.0	88.0 ± 1.3	86.0–100.0	4.31	4.9
2.	*G. herbaceum* subsp. *pseudoarboreum*	22	10	20.0	18.0	2.0	90.0 ± 1.2	85.0–100.0	3.94	4.3
3.	*G. herbaceum* subsp. *pseudoarboreum* f. * harga*	42	10	16.0	14.0	2.0	90.0 ± 2.4	80.0–100.0	7.63	8.4
4.	*G. herbaceum* subsp. *euherbaceum* (cv. А-256)	49	10	16.0	15.0	1.0	91.0 ± 2.5	75.0–100.0	8.15	8.9
Australian wild species										
5.	*G. australe*	41	10	11.0	10.0	1.0	94.5 ± 1.5	90.0–100	4.8	5.1
6.	*G. nelsonii*	45	10	13.0	12.0	1.0	95.0 ± 1.3	91.0–100	4.2	4.4
7.	*G. bickii*	43	10	9.0	8.0	1.0	93.0 ± 1.9	86.0–100	6.12	6.6

**Table 2:** Boll- and seed-setting rates in the hybrids of the crosses between *G. herbaceum* × Australian wild species.

No.	Hybrid Combinations	Number of Crosses	Number of Obtained Hybrid Bolls	Boll-Setting Rate, %	Seed-Setting Rate, %			
					$\bar{x}\pm S\bar{x}$	Range	S	V
1.	*G. herbaceum* subsp. *africanum × G. australe*	38	-	-	-	-	-	-
2.	*G. herbaceum* subsp. *africanum × G. bickii*	24	-	-	-	-	-	-
3.	*G. herbaceum* subsp. *africanum × G. nelsonii*	9	1	11.1	-	-	-	-
4.	*G. herbaceum* subsp. *pseudoarboreum × G. nelsonii*	47	10	21.3	62.4 ± 4.6	42.1–86.4	23.4	7.4
5.	*G. herbaceum* subsp. *pseudoarboreum × G. australe*	18	5	27.8	60.4 ± 5.3	42.1–77.8	12.7	21.1
6.	*G. herbaceum* subsp. *pseudoarboreum × G. bickii*	51	10	19.6	66.7 ± 4.7	47.6–85.0	22.3	7.1
7.	*G. herbaceum* subsp. *pseudoarboreumf. harga × G. nelsonii*	22	6	27.3	54.3 ± 0.8	52.0–53.9	2.1	3.8
8.	*G. herbaceum* subsp. *pseudoarboreum f. harga × G. bickii*	11	2	18.2	51.2 ± 3.8	47.4–55.0	5.4	10.5
9.	*G. herbaceum* subsp. *frutescens × G. australe*	40	-	-	-	-	-	-
10.	*G. herbaceum* subsp. *frutescens × G. nelsonii*	109	2	1.8	62.6 ± 7.5	55.2–70.0	10.5	16.7
11.	*G. herbaceum* subsp. *frutescens × G. bickii*	108	-	-	-	-	-	-
12.	*G. herbaceum* subsp. *euherbaceum* (cv. А-256) *× G. bickii*	19	1	5.3	50.0	50.0	-	-
13.	*G. herbaceum* subsp. *euherbaceum* (cv. А-256) *× G. nelsonii*	9	6	66.7	41.0 ± 3.8	30.0–61.5	29.4	9.3
14.	*G. herbaceum* subsp. *euherbaceum* (cv. А-256) *× G. australe*	36	1	2.7	50.0	-	-	-

**Table 3:** Heredity and variability of the vegetative growth duration in the F_1_C and F_2_C polyploid hybrids and parental lines.

No.	Plant Samples	Vegetative Growth Duration, Days					
		$\bar{x}\pm S\bar{x}$	Range	S	V%	hp	h^2^
Parental lines (*G. herbaceum* subspecies)							
1.	*G. herbaceum* subsp. *africanum*	142.9 ± 0.4	139–146	1.9	1.38	-	-
2.	*G. herbaceum* subsp. *pseudoarboreum*	121.3 ± 0.4	119–123	1.5	1.2	-	-
3.	*G. herbaceum* subsp*. pseudoarboreum* f*. harga*	126.2 ± 0.4	123–128	1.9	1.5	-	-
4.	*G. herbaceum* subsp*. frutescens*	117.2 ± 0.4	116–119	1.3	1.1	-	-
5.	*G. herbaceum* subsp. *euherbaceum* (cv. А-256)	137.1 ± 0.4	135–139	1.6	1.2	-	-
Parental lines (Australian wild species)							
6	*G. australe*	133.6 ± 0.3	132–135	1.1	0.8	-	-
7	*G. nelsonii*	135.3 ± 0.3	134–138	1.5	1.1	-	-
8	*G. bickii*	135.5 ± 0.4	133–138	1.8	1.4	-	-
F_1_С polyploid hybrids							
9	*G. herbaceum* subsp. *frutescens* × *G. nelsonii*	127.5 ± 0.4	126–130	1.4	1.1	–0.13	-
10	*G. herbaceum* subsp. *euherbaceum* (cv. А-256) × *G. nelsonii*	128.1 ± 0.5	125–130	1.9	1.5	–0.9	-
F_2_С polyploid hybrids							
11	*G. herbaceum* subsp. *frutescens* × *G. nelsonii*	116 ± 1.05	113–123	3.3	2.9	0.13	–0.1
12	*G. herbaceum* subsp. *euherbaceum* (cv. А-256) × *G. nelsonii*	127.9 ± 1.7	123–137	5.3	4.2	9.2	–0.1

**Table 4:** Rates of infestation (%) of genotypes by cotton aphids and whiteflies.

No.	Genotypes	Х ± Sх		Range		S		V (%)	
		Aphids	Whiteflies	Aphids	Whiteflies	Aphids	Whiteflies	Aphids	Whiteflies
1	ssp. *frutescens*	7.3 ± 0.38	8.4 ± 0.22	5.5–8.7	7.4–9.2	1.07	0.62	14.63	7.47
2	ssp. *euherbaceum* (cv. A-256)	7.9 ± 0.46	8.9 ± 0.32	6.0–9.3	7.7–10.1	1.29	0.91	16.25	10.2
3	*G. nelsonii*	6.0 ± 0.37	6.3 ± 0.22	4.7–7.7	5.2–7.2	1.04	0.62	17.36	9.83
4	ssp. *euhirsutum* (cv. Ravnak-1)	22.1 ± 0.32	23.9 ± 0.43	21.0–23.3	22.5–25.5	0.91	1.23	4.08	5.14
5	F_1_ (ssp. *frutescens* × *G nelsonii*)	6.5 ± 0.28	6.4 ± 0.22	5.3–7.5	5.9–7.7	0.79	0.62	14.36	9.63
6	F_1_ (ssp. *euherbaceum* × *G. nelsonii*)	6.55 ± 0.42	6.8 ± 0.35	4.9–8.0	5.7–8.0	1.19	0.98	18.16	14.42
7	F_1_C (ssp. *frutescens* × *G.nelsonii*)	5.56 ± 0.33	5.4 ± 0.27	4.3–6.7	4.4–6.5	0.92	0.75	16.59	13.95
8	F_1_C (ssp. *euherbaceum* × *G. nelsonii*)	5.16 ± 0.19	5.8 ± 0.22	4.3–6.0	4.9–6.6	0.54	0.62	10.45	10.76
9	F_1_ (ssp. *frutescens* × *G.nelsonii*) × cv.Ravnak-1	6.56 ± 0.30	7.5 ± 0.26	5.7–7.7	6.5–8.6	0.84	0.73	12.77	9.8
10	F_1_ (ssp. *euherbaceum* × *G. nelsonii*) × cv. Ravnak-1	8.00 ± 0.30	10.6 ± 0.44	6.4–8.7	8.8–12.4	0.84	1.24	10.52	11.67

**Table 5:** Analysis of tetrads in the studied cotton samples.

No.	Plant Samples	Total Number of Spores	Meiotic Index, %	Micronuclear Tetrads, %	Polyads, %
Parental forms					
1	*G. herbaceum* subsp. *frutescens*	787	94.2 ± 0.8	-	0.7 ± 0.3
2	*G. herbaceum* subsp. *euherbaceum* (cv. А-256)	907	89.8 ± 1.01	2.65 ± 0.5	2.7 ± 0.5
3	G. *nelsonii*	1003	95.6 ± 0.9	-	0.1 ± 0.02
F_1_ hybrids					
4	*G. herbaceum* subsp. *frutescens* × *G. nelsonii*	547	57.8 ± 2.1	20.3 ± 1.7	5.5 ± 0.9
5	*G. herbaceum* subsp. *euherbaceum* (cv. А-256) × *G. nelsonii*	1011	78.6 ± 1.3	6.9 ± 0.8	2.5 ± 05
F_1_C allotetraploid hybrids					
6	*G. herbaceum* subsp. *frutescens* × *G. nelsonii*	380	46.8 ± 2.6	37.6 ± 2.5	1.8 ± 0.7
7	*G. herbaceum* subsp. *euherbaceum* (cv. А-256) × *G. nelsonii*	378	47.1 ± 1.9	36.7 ± 2.5	1.9 ± 0.7

**Table 6:** Development of cotton pollens in the F_1_ and F_1_C hybrids.

No.	Hybrid Combinations	Total Number of Pollens	Pollen Fertility, %			
			$\bar{x}\pm S\bar{x}$	Range	S	V %
F_1_ hybrids						
1	*G. herbaceum* subsp. *frutescens × G. nelsonii*	1227	4.56 ± 0.35	0.75–7.3	0.60	0.4
2	*G.nelsonii × G. herbaceum* subsp. *frutescens*	1356	4.72 ± 0.33	0.75–7.54	0.58	0.3
3	*G. herbaceum* subsp. *pseudoarboreum × G. nelsonii*	879	6.14 ± 0.66	5.51–8.71	0.81	0.7
4	*G. nelsonii × G. herbaceum* subsp. *pseudoarboreum*	1112	5.67 ± 0.48	5.28–7.53	0.69	0.5
5	*G. herbaceum* subsp. *pseudoarboreum × G. australe*	1334	2.25 ± 0.17	1.96–2.87	0.41	0.2
6	*G. herbaceum* subsp. *pseudoarboreum* f. *harga × G. nelsonii*	512	8.79 ± 1.57	5.18–9.98	1.25	1.3
7	*G. nelsonii × G. herbaceum* subsp. *pseudoarboreum* f. *harga*	1660	7.29 ± 0.41	7.13–8.67	0.64	0.4
8	*G. herbaceum* subsp. *euherbaceum* (cv. А-256) *× G. australe*	685	4.82 ± 0.67	3.18–7.66	0.82	0.7
9	*G. herbaceum* subsp. *euherbaceum* (cv. А-256) *× G.nelsonii*	721	7.07 ± 0.91	6.98–11.1	0.95	0.9
10	*G. herbaceum* subsp. *euherbaceum* (cv. А-256) *× G. bickii*	825	4.73 ± 0.55	3.94–5.34	0.74	0.6
F_1_C allotetraploid hybrids						
11	*G. herbaceum* subsp. *frutescens × G. nelsonii*	2307	47.2 ± 1.04	43.1–51.7	1.08	1.04
12	*G. herbaceum* subsp. *euherbaceum* (cv. А-256) *× G. nelsonii*	2306	54.1 ± 1.04	46.8–66.5	1.07	1.04

## Data Availability

Data are contained within the article and the Supplementary Materials.

## Supplementary Materials

The following supporting information can be downloaded at: https://www.mdpi.com/article/10.3390/plants14111620/s1, Table S1. The list of cotton species and subspecies used in this study; Table S2. SSR marker polymorphism: PIC and heterozygosity values (Refserences [35,36,37,38,39,40,41,42,43,44,45,46,47,48,49,50,51,52,53,54,55,56,57,58,59,60,61,62,63,64,65,66,67,68,69] are cited in the Table S2); Figure S1. The scheme of obtaining allotetraploid cotton genotypes; Figure S2. Normal and abnormal tetrads in F_1_C hybrids of *Gossypium herbaceum* and *G. nelsonii*; Figure S3. Pollen fertility of cotton species and hybrids.
