# Supplementary material for: Development and Characterization of Synthetic Allotetraploids Between Diploid Species Gossypium herbaceum and Gossypium nelsonii for Cotton Genetic Improvement
Source: Plants (Basel). 2025 May 26;14(11):1620. doi: 10.3390/plants14111620 (PMC12158148; doi:10.3390/plants14111620)
Supplement: Supplementary file 1 [file plants-14-01620-s001.zip › plants-3626189-supplementary.pdf]

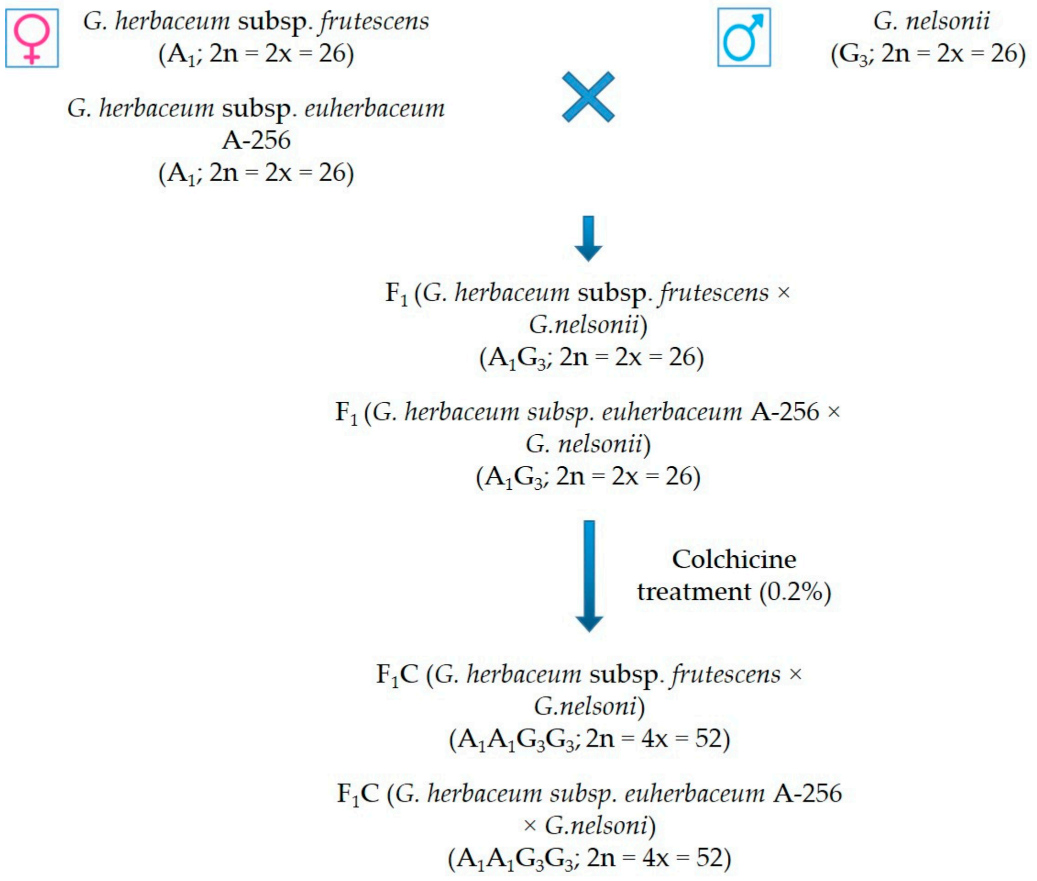

Figure S1. The scheme of obtaining allotetraploid cotton genotypes.

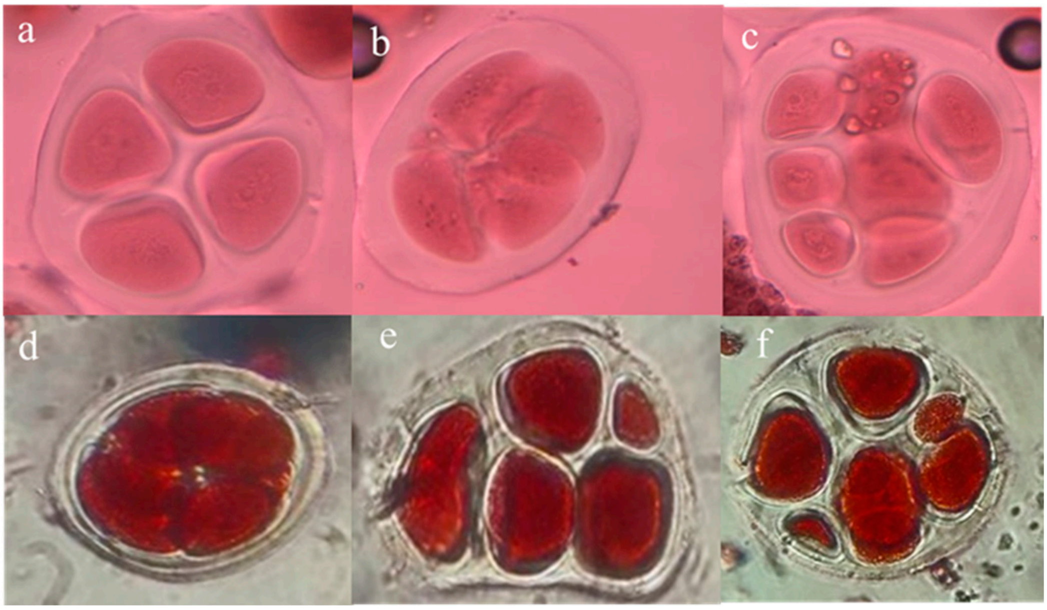

Figure S2. Normal and abnormal tetrads in F_1C hybrids of *Gossypium herbaceum* and *G. nelsonii*. a) normal tetrads; b, e) tetrads with micronuclei (abnormal); c) heptads; d) pentads with micronuclei; f) tetrads with two micronuclei.

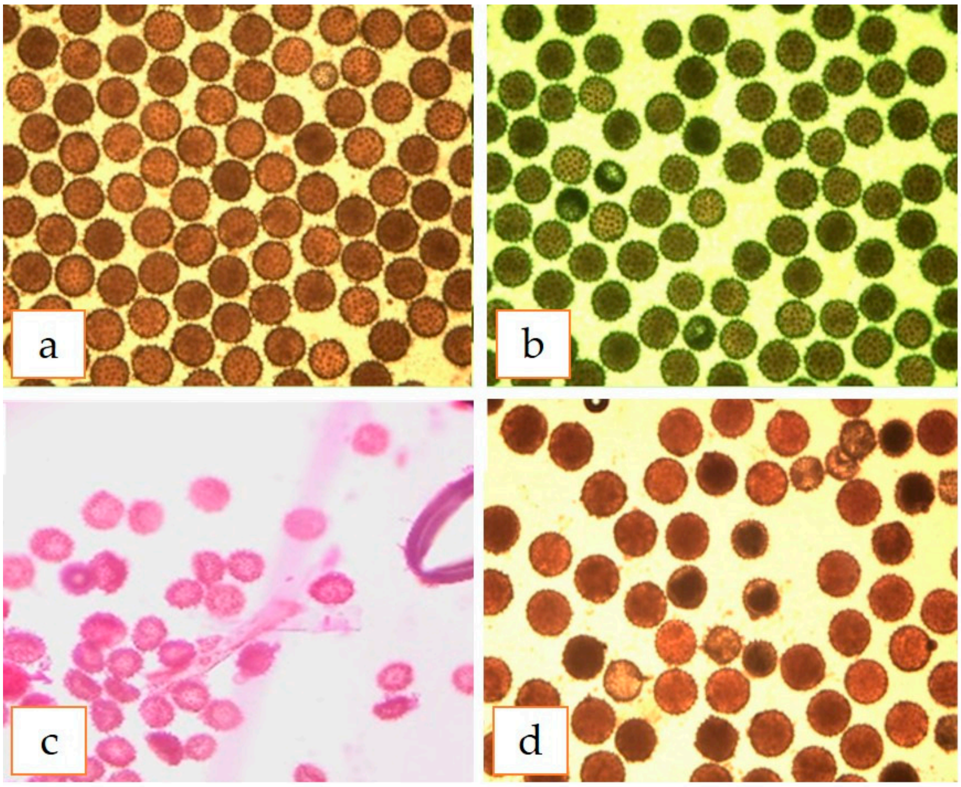

Figure S3. Pollen fertility. a) *G. herbaceum* subsp. *frutescens* (98,6±0,34); b) *G. nelsonii* (96,36±0,4); c) F_1 (*G. herbaceum* subsp. *frutescens* × *G. nelsonii*) (4,56±0,35); d) F_1C (*G. herbaceum* subsp. *frutescens* × *G. nelsonii*) (47.2±1.04).

Table S1. The list of cotton species and subspecies used in this study.

№	Name of subspecies and variations	Genome	Origin	Collection
1	*G. herbaceum* subsp. *africanum*	A_1	Angola, Northern Namibia, Mozambique, Tanzania	IGPEB*
2	*G. herbaceum* subsp. *pseudoarboreum*	A_1	Mauritania, Senegal, Guinea, Liberia, Mali, Togo, Benin, Nigeria, Ethiopia, Southeast Arabia	IGPEB
3	*G. herbaceum* subsp. *pseudoarboreum* f. *harga*	A_1	Sudan	IGPEB
4	*G. herbaceum* subsp. *frutescen*s	A_1	Africa, Sudan, Northern Nigeria, Chad, Zambezi, Upper Nile, Iran, Iraq, India.	IGPEB
5	*G. herbaceum* subsp. *euherbaceum,* (cv. A-256)	A_1	Chad, Sudan, Morocco, Libya, Egypt, Arabia, Iraq, Iran, Afghanistan, China, India, Pakistan	IGPEB
6	*G. bickii* Prokh.	G_1	Georgina River Banks, Queensland, Central Australia	IGPEB
7	*G. australe* F. Muell.	G_2	Central and northern Australia, mainly distributed in desert zones	IGPEB
8	*G. nelsonii* Fryxell.	G_3	Northwestern, northern and northeastern Australia.	IGPEB
9	Cv. Ravnak-1	AD_1	Uzbekistan	CGB**
10	Cv. Ravnak-2	AD_1	Uzbekistan	CGB
11	Cv. Baraka	AD_1	Uzbekistan	CGB

* Institute of Genetics and Plant Experimental Biology; ** Cotton Genomics and Bioinformatics

Table S2. SSR Marker Polymorphism: PIC and Heterozygosity Values

No.	Marker	Forward primer (5'-3')	Reverse primer (5'-3')	Molecular weight (bp)	PIC	He	Associated trait	Chr. No*	Reference
1	DPL0068	gttcaacaggtctgtaccagttcc	gcaaatgatctctgccctgtaa	200/220	0.339	0.434	Flower and boll period (FBP)/growth period (GP)	D08	[35]
2	DPL0131	acatacgggttgaaatgtactcct	atgaatgcagatcattacgcct	190/210	0.275	0.329	Fiber quality	D11	[36]
3	DPL0181	aaaccctaacgtaattagcttctcg	cccagtgaaaggtcttaagaaagaa		0.278	0.334			
4	DPL0244	gaggtggaagtggagataaagatg	catctacatcatgagaccgaatgt		0.253	0.298			[37]
5	DPL0249	acagagctatgggaaatcatggta	tgtactgcaaattgctgctaagac		0.253	0.298		A18	[38]
6	DPL0280	agtgtaccactaagtcttcgagcc	gaatcattactgtcaccttgagca		0.305	0.375			
7	DPL0300	gaaatattgcttgtctgcttcagg	gggtcaaaccacgactttattaga		0.239	0.277		A15	[38]
8	DPL0461	cccaacctctgatcacctctatac	ctttcccactcgtaacaaggtaaa		0.341	0.444		A24	[38]
9	DPL0473	cgttacaggcgtaactaaagaggt	atgtatatgacattgagtggctgc	180/200	0.307	0.379	Resistance to FOW	D02	[39]
10	DPL0500	agtctctaaacctgactcctgcac	tttcatgcagtacatcgtacacttc		0.253	0.298			
11	DPL0530	agacttacttaaaggcaccattcg	gcagactcttctggtgtaacagtg	220/230	0.269	0.320	FM/FL/FS/FU	A09	[40]
12	DPL0590	gatttactttaaggaggcgaaac	aaaggtacactcatgcgactgac	220.250	0.239	0.277	Fiber quality	A06	[36]
13	DPL0665	gcagaaacccacctaatttaagaag	gtagaggttggagcacttgtcatt	130/135/140/155/200	0.310	0.384	Resistance to Verticilium wilt	A06/D 12	[41]
14	DPL0681	aaatggacaaattcgactccc	ttcgactcgactgaatcgtaacta		0.329	0.416		A06	[38]
15	DPL0852	gttccaaatcaatctcgtgt	ggctgttacagatcaaactccc	150/170	0.268	0.320	Fiber quality	A07	[42]
16	Gh034	cccttttgttatctaacttctgttactcctaac	ccttttgtttagctcttctatacttgaattcc	135/145/150	0.268	0.320	Lint cotton yield/micronaire	A13	[43]
17	Gh056	tccattagacaaagttttctaaagttc	tgagacttccaaccagatacag	80	0.268	0.320	Fiber quality	D07	[44]
18	Gh064	gagaagccaatcccatttaaga	gatagtgcatctctagagaaggaca	110/130	0.339	0.434	Fiber quality	A12	[45]/[46]
19	Gh109	caagaaggaaatggctgaattg	cagacaccagctgttgcc		0.228	0.263			
20	Gh110	accatcccaaagaatcatcctc	actaaaaccaaggcaataaagtg	180/190	0.233	0.269	Fiber quality	D10	[46]
21	Gh132	tcatggaacaccaaagttgga	acatgatagattattcagcaatgca	140/145/150/155/170	0.231	0.267	Reniform nematode-resistant	D11	[47]
22	Gh158	ggtgctcatgatgatgctg	gagtcaatgacaagaggatcacc		0.297	0.363			[38]
23	Gh243	cagaaggttatgcaaacaacatgca	ctaaactctctctgctgtgttcc	75/105/165	0.375	0.500	Resistance to FOW	A12	[48]

24	Gh247	cttcttccgccacgtaagtcc	cagcctaaccaagaacccaatcg	130/135/145	0.241	0.280	Micronaire	A09	[49]
25	Gh262	gtcaacaaccttaaaattgccataggt	tggactaccaattaacttgccacg	150/185/200	0.207	0.235	Resistance to FOW	A05	[39]
26	Gh277	tactaaaaccaaggcaataaagtga	caccaccttccatatatcttgctc	100/110/115	0.331	0.418	Short fiber	D10	[32]
27	Gh388	catcatcatcgtcgtcgccg	gcaatggaagctttcgtctctttc	150/415/615/930/1210	0.268	0.320	Fiber quality	A05	[50]
28	Gh433	taccacattggatgtttgcaaaccc	atagcaaactggaatcactccaagc	115/170/185	0.268	0.320	Resistance to Verticilium wilt	A06	[51]
29	Gh591	gatttgaaaactggaggcatctcc	tcggttaccaccaatttaaccagc	100/105/140/150	0.295	0.360	Fiber length	A06	[52]
30	Gh592	ttgttatctaacttctgttactcctaac	ttgtttagctcttctatacttgaattcc	50/100/110	0.289	0.351	Fiber quality	A13	[53]
31	HAU0091	cttcaaggagtcagatttgc	ttaaatcctcaccgagatgg	320/325/335/340/350	0.299	0.367	Resistance to Verticilium wilt	D04	[51]
32	HAU0423	cgatggaagaaaaatcgaaa	atatttcgaccaaggagtcg	170/190	0.282	0.341	Resistance to Verticilium wilt	A11	[49]
33	HAU0483	cgaggtggatccctataaaa	tttcggtgaattgagaaaca		0.304	0.375			
34	HAU0639	tttgaacgaacacattacgg	atgggtttttaccagagcag	75/80	0.282	0.341	Fiber quality	A11	[46]
35	HAU0878	tcattcctgaaacccaaaat	ctaacaggggtgacataggg	120/140	0.239	0.277	Fiber quality	A05	[54]
36	HAU0921	tcaagcaaaatatggggttt	ggtaaacaggtgggtgagtt		0.318	0.396			
37	HAU1081	tgtctcccgtactcagtgaa	ggctatggggttacaatcag	80/85	0.207	0.235	Fiber quality	D12	[46]
38	HAU1264	ctctctctctcaaacacctcct	caaccccatatcctcttttg	60/65/70/75	0.304	0.375	Resistance to FOW	D03	[55]
39	HAU1314	gaaaagccctttaccaacaa	tcagctctcctatctcacctc	65/70/75	0.268	0.320	Fiber quality	A10	[56]
40	HAU1332	ttggcattgagtacgcttta	ttgcttcattcgtagtgcat	210	0.282	0.329	Fiber quality	A04	[49]/[57]/[58]
41	HAU1371	ggggtgtttggcttattaaa	agaagcgatatgaggtccag	305/310/315	0.304	0.375	Fiber elongation	A06	[37]/[57]
42	HAU1399	atgacaatttcagctcacga	ccaattcacacgtatttgga	85/90	0.282	0.341	Fiber quality	A07	[46]
43	HAU1434	ggttagaccaggagcaaatg	tcactgagatttggagctga		0.259	0.307			
44	HAU1455	gaaggtcaatccaaggtgtc	tggctcctagttgtttttcc		0.304	0.375	FL	D02	[49]
45	HAU1460	aaaattcaccccctcacc	ggctgatgctgaactaacct		0.345	0.444			[37]
46	HAU2583	accaagactggggttggggt	tggatgccttgcctccctct		0.200	0.226			
47	HAU2625	ctgccttgctcctgcacctt	ggggtaaacaggcgggtgag	105/110	0.375	0.500	Fiber quality	D06	[57]/[58]
48	HAU2768	agtgccatctgcttcggctc	tgtgaacaatgaaagtctgaccct	110/115/125	0.295	0.360	Fiber quality	A06	[57]/[58]
49	JESPR095	gcttttctcgtagacgtatg	gcatatttatataccaagtccctc	100	0.295	0.360	-	A09	[59]
50	JESPR114	cccaacctctgatcacctctatac	ctttcccactcgtaacaaggtaaa	90/95	0.295	0.360	Fiber quality	D09	[59]
51	JESPR156	gccttcaatcaattcatacg	gaaggagaaagcaacgaattag	95/ 130/150/170/175/180			Resistance to FOW	D02	[55]

52	BNL0226	ttattctcacagccggaacc	ttcaccctctcgcttctcat	190/200/205	0.181	0.201	Fiber quality	AD03	[59]
53	BNL0530	cgtaggatggaaacgaaagc	gccacacttttccctctcaa	140/200	0.298	0.364	Fiber quality	D04	[60]
54	BNL0598	tatctccttcacgattccatcat	aaaagaaaacagggtcaaaagaa	125	0.318	0.396	Fiber quality	A12	[61]
55	BNL1167	acggcgttacaaggcattac	acttagtttctcaaaaaaaaaatgc	200	0.345	0.444	Fiber quality	A04	
56	BNL1231	taataaaagggaaaggaaagagtt	tatggctctagaatattccctcg	185/190/195/205	0.345	0.444	Salt tolerance_RSOD	A03	[62]
57	BNL1421	tgaagatttggaggcaattg	gaaatcaagcctcaattcgg	190/210	0.318	0.396	Lint cotton yield/micronaire	A13	[44]
58	BNL3255	gacagtcaaacagaacagatatgc	ttacacgacttgttcccacg	300/320/400/450/470/500	0.253	0.297	Fiber quality/VW	A 04	[63]
59	fBNL3436	aacatagcctaccattgccg	ttgtttgccaaatttgaagc	130/180	0.268	0.320	Fiber length/fiber micronaire	D25	[62]/[64]
60	BNL3601	ttccgttgatggaaattgaa	acaagaatgcgtgtgtctgc	150/165	0.304	0.375	Maturity/cell wall thickness	A05	[65]
61	BNL1694	cgtttgttttcgtgtaacagg	tggtggattcacatccaaag	100/105/110/115	0.268	0.320	Fiber quality/Salt tolerance_RPH/RGR/RGP	D16/A07	[44]/[62]/[66]
62	BNL3171	gaaaaattgaggaaggacatacg	ggccacaaccgaatttactg	130	0.307	0.379	Fiber quality	D11	[61]
63	BNL1604	agagggagtaaagatttgggg	tccagttctttttgccttgg	60/65/70/75	0.304	0.375	Fiber quality	A07	[66]/[67]
64	BNL3347	agactgacatgcagcttcca	atcttaattttgagtataggatagggg	150/160/200/230/240/600	0.345	0.444	Fiber quality	A05	[60]
65	BNL3359	ttgttgttgggaatgatgga	tgacccttcaccgactttct	205/210	0.335	0.427	Fiber quality	D06/A06	[61]
66	BNL3424	tgtgccgtctcaaaatgaag	aagaccaatctgttgccagc	160/180	0.367	0.485	Relative malondialdehyde	D03	[62]
67	BNL3452	tgtaactgagcagccgtacg	gccaaagcagagtgagatcc	190	0.278	0.334	Relative malondialdehyde	D19/A05	[62]
68	BNL3594	agggattttgattgttgtgc	tgaattcaaaacaaatgttagcc	160/170/210	0.261	0.308	Salt tolerance/salt_RMDA/SY (seed cotton yield)/BN (bolls per plant)	A06	[44]/[62]/[66]
79	BNL3650	tcgatttccttatttgatttctg	aatttgtccagattcattcttca	370/375	0.343	0.440	FL/FE	A06	[66]
70	NAU1157	gagtttggttctgggttgag	gatccttttcatctcctcca	240/250	0.342	0.321	Resistance to FOW	D03	[68]
71	NAU1355	atctgtttacgccactctcc	ccagcctttgacatttttct	260/265	0.324	0.412		D08	
72	NAU1052	cgcagataaaggatggattt	agagctggaggacataacaaa	170/200	0.357	0.431		D11	
73	NAU3093	gtcttgaaccggaacttgat	tcctgttgaacaccaaagtg	275	0.298	0.392		A04	

74	BNL3792	ttcgagatcccctgttctga	catattccagtcaaaccaaacg	240/500	0.318	0.396	Relative plant height	A08	[62]/[69]
